# Pre‐ and postzygotic mechanisms preventing hybridization in co‐occurring species of the *Impatiens purpureoviolacea* complex

**DOI:** 10.1002/ece3.8382

**Published:** 2021-11-24

**Authors:** Stefan Abrahamczyk, Michaela Jandová, Zuzana Líblová, Steven B. Janssens, Tomáš Dostálek, Norbert Holstein, Eberhard Fischer

**Affiliations:** ^1^ Nees Institute for Biodiversity of Plants University of Bonn Bonn Germany; ^2^ Institute of Botany The Czech Academy of Sciences Průhonice Czech Republic; ^3^ Department of Botany Faculty of Science Charles University Prague Czech Republic; ^4^ Meise Botanic Garden Meise Belgium; ^5^ Department of Biology Institute of Botany and Microbiology KU Leuven Leuven Belgium; ^6^ Natural History Museum London UK; ^7^ Institut für Integrierte Naturwissenschaften – Biologie Universität Koblenz‐Landau Koblenz Germany

**Keywords:** Albertine Rift, bird pollination, chromosome number, evolution, hybridization, insect pollination, polyploidization

## Abstract

In the species‐rich genus *Impatiens*, few natural hybrids are known, even though closely related species often occur sympatrically. In this study, we aim to bridge the gap between micro‐ and macro‐evolution to disentangle pre‐ and postzygotic mechanisms that may prevent hybridization in the *Impatiens purpureoviolacea* complex from Central Africa. We analyzed habitat types, species distribution, pollination syndromes, pollinator dependency, genome sizes, and chromosome numbers of seven out of the ten species of the complex as well as of one natural hybrid and reconstructed the ancestral chromosome numbers of the complex. Several species of the complex occur in sympatry or geographically very close to each other. All of them are characterized by pre‐ and/or postzygotic mechanisms potentially preventing hybridization. We found four independent polyploidization events within the complex. The only known natural hybrid always appears as single individual and is self‐fertile. But the plants resulting from self‐pollinated seeds often die shortly after first flowering. These results indicate that the investigated mechanisms in combination may effectively but not absolutely prevent hybridization in *Impatiens* and probably occur in other genera with sympatric species as well.

## Graphical abstract

Several species of the complex occur in sympatry or geographically very close to each other. All of them are separated by pre‐ and/or postpollination hybridization barriers. We found four independent polyploidization events within the complex.

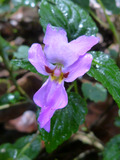

## 1 INTRODUCTION

Hybridization is a mechanism that can facilitate but also reduce speciation and even merge the gen pools of two existing species (Abbott et al., 2013; Soltis & Soltis, 2009). However, surprisingly few natural hybrids are known in some species‐rich genera with sympatric species, such as *Impatiens* (Balsaminaceae), *Astragalus* (Fabaceae), *Pedicularis* (Orobanchaceae), and *Ixora* (Rubiaceae; e.g., Bartha et al., 2013; Grey‐Wilson, 1980a; Liang et al., 2018; Mouly et al., 2009) even though artificial hybrids are commonly produced, at least in *Impatiens*, sharing flower traits of both parents (Morgan, 2007). Probably, the evolution in these genera included the development of effective mechanisms to prevent hybridization. In general, two classes of such mechanisms can be distinguished but often a combination of different barriers exists (Seehausen et al., 2014; Sobel & Chen, 2014): First, prezygotic mechanisms include for example geographical or habitat isolation as well as adaptation to different groups of pollinators, temporal variation in flowering time, and different reproductive systems (Arnold, 1997; Bradshaw & Schemske, 2003; Lumaret et al., 1987; Neri et al., 2017). Second, postzygotic mechanisms include for example inhibition of pollen tube growth, failure of normal seed development, and a reduced seed fertility and seedling fitness among other mechanisms (Lafon‐Placette & Köhler, 2016; Lee et al., 2008; Merlin & Grant, 1986). Abortion or reduced fertility of seeds is often caused by failure of endosperm development, for example, if mother and father plants have unequal chromosome numbers (Birchler, 2014; Husband & Sabara, 2004; Ramsey & Schemske, 1998). Especially in species‐rich lineages, highly diverse chromosome numbers often occur in combination with differences in genome sizes (e.g., Cai et al., 2019; Escudero et al., 2012; Han et al., 2020; Mota et al., 2016). Additionally, differences in other characters, such as habitat type and pollinator group (Glennon et al., 2012; Sobel et al., 2010), commonly exist in these species. All of these differences represent effective mechanisms preventing hybridization in many groups of plants (Birchler, 2014; Sobel et al., 2010), but few studies exist analyzing different mechanisms in larger clades with co‐occurring species, even though such studies would provide deep insights into the evolution of these clades.

The species‐rich genus *Impatiens* (Balsaminaceae; >1000 species) is an ideal group to study mechanisms potentially preventing hybridization (Janssens et al., 2009). It occurs mostly in the humid forests of the tropics and subtropics in Africa and Asia (Grey‐Wilson, 1980a). In these habitats often several, sometimes even closely related *Impatiens* species occur sympatrically (e.g., Janeček et al., 2015; Kato et al., 1991; Ruchisansakun et al., 2016). However, until now only few natural hybrids have been found (Fischer et al., 2021; Grey‐Wilson, 1980b, 1980c,1980b, 1980c; Tsukaya, 2004). Most of these hybrids occur in disturbed places in small to medium‐sized populations (Grey‐Wilson, 1980b, 1980c,1980b, 1980c). Furthermore, the proposed hybrid origin of several *Impatiens* species (Grey‐Wilson, 1980b, 1980c,1980b, 1980c) has never been demonstrated and seems unlikely based on the recent molecular analyses on the genus (e.g., Janssens et al., 2009) and a few hybridization studies (e.g., Merlin & Grant, 1986; Ornduff, 1967; Tsukaya, 2004).

Due to the rare nature of hybrids but large numbers of co‐occurring species, we can conclude that strong mechanisms preventing hybridization must exist in *Impatiens*. However, mostly prezygotic mechanisms have been studied in *Impatiens*: Besides isolation by geography and habitat type (Merlin & Grant, 1986) a common element preventing hybridization in *Impatiens* are switches between pollinator groups in closely related *Impatiens* species (Grey‐Wilson, 1980a; Janeček et al., 2015; Lozada‐Gobilard et al., 2019). Additionally, Ruchisansakun et al. (2016) demonstrated that within the same habitat a group of species with asymmetric flowers—all pollinated by the same assemblage of bees—do not hybridize because each species deposits its pollen on different parts of the bee´s bodies.

In addition to the mentioned prezygotic mechanisms, also postzygotic mechanisms must exist in *Impatiens*. For example, *Impatiens glandulifera* and *I. balfourii*, two neophytic species occurring side by side in southern Europe, get visited by the same species of bumblebees (Ugoletti et al., 2013). Regular occurrence of heterospecific pollen on the stigmas inducing seed formation is documented (Ugoletti et al., 2013). However, no hybrids are known because hybrid seeds mostly fail to germinate in crossing experiments (Ugoletti et al., 2013). Consequently, strong genetic barriers probably exist between these distantly related species, preventing hybridization. Differences in chromosome numbers are likely the reason for unsuccessful hybridization between previously mentioned *I. glandulifera* (2*n* = 18) and *I. balfourii* (2*n* = 14; Song et al., 2003). Similar to this example different chromosome numbers probably occur in many other sympatric *Impatiens* species because a large diversity of chromosome number is known within *Impatiens* (2*n* = 6 to 2*n* = 200 with a majority of species with 2*n* = 14 to 2*n* = 20; Jeelani et al., 2010; Song et al., 2003). However, chromosome number evolution has not systematically been studied in *Impatiens*.

A promising group to study mechanisms potentially preventing hybridization in closely related species is the *Impatiens purpureoviolacea* complex endemic to the mountain rainforests of the northwestern Albertine Rift Valley (in Rwanda, Burundi, and the Democratic Republic of the Congo). It originated in the Pliocene and started diversifying during the transition of Pliocene and Pleistocene, possibly triggered by an increased mountain uplifting and volcanic activity in the Albertine Rift (Fischer et al., 2021). The clade consists of ten species that partly occur sympatrically or geographically close to each other. Most of them show a butterfly/long‐tongued bee pollination syndrome with long, filiform, strongly enrolled flower spurs (Abrahamczyk et al., 2017; Fischer et al., 2021). Only two species have bucciniform spurs and are likely pollinated by birds (Fischer et al., 2021). Even though several species of the *Impatiens purpureoviolacea* complex occur sympatrically or geographically close to each other and flower simultaneously only a single, rarely occurring hybrid is known (Fischer et al., 2021). Therefore, we can assume that strong mechanisms preventing hybridization exist.

Here, we analyze mechanisms possibly preventing hybridization of seven out of ten species and one natural hybrid from the *Impatiens purpureoviolacea* complex. We study prezygotic (habitat types, geographical distribution, pollination syndromes, and pollinator dependency) and postzygotic (chromosome numbers and genome sizes) mechanisms that may prevent hybridization with special focus on the sympatric/geographically close species and put the traits into a phylogenetic context. Specifically, we form the following hypotheses:

1. Pre‐ and postzygotic mechanisms exist in the *Impatiens purpureoviolacea* complex that may prevent hybridization.
2. Co‐occurring species are always separated by at least one pre‐ or postzygotic mechanism.

## 2 MATERIALS AND METHODS

### 2.1 Plant material

**FIGURE 1:**
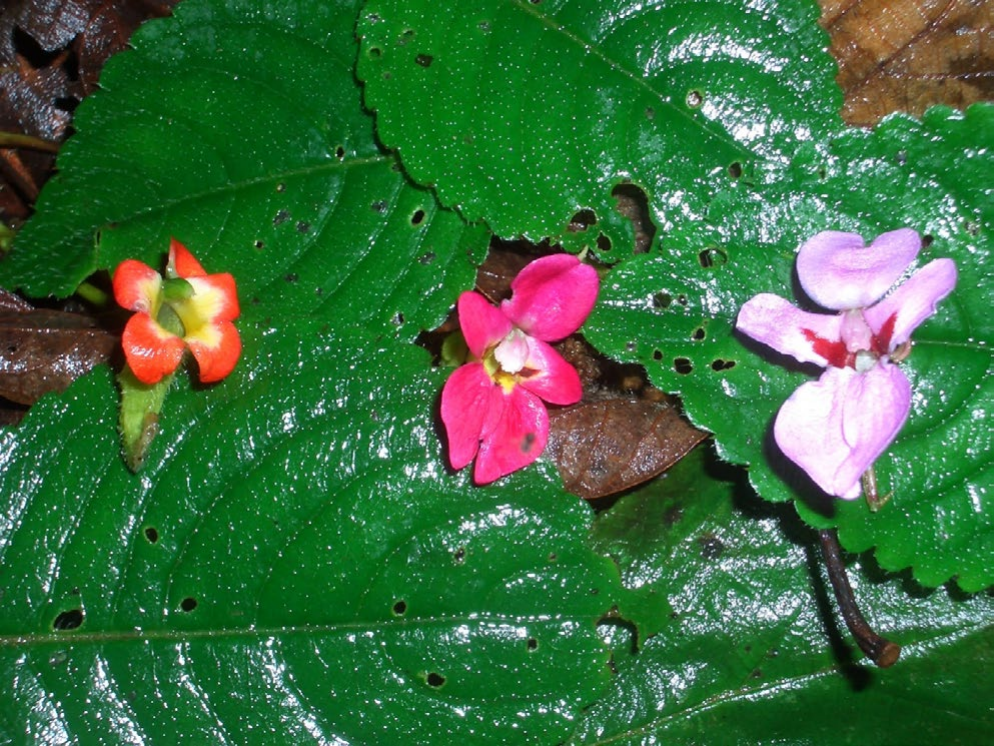
*Impatiens* × *troupinii*. Flower, frontal view (middle), with flowers of its parental species *Impatiens gesneroidea* (left) and *I*. *purpureoviolacea* (right); Captured by E. Fischer

This study benefits from the extensive sampling by Eberhard Fischer since 1984 resulting into a recent revision of the *Impatiens purpureoviolacea* complex (Fischer et al., 2021). Seven out of ten species and one natural hybrid (Figure 1) are studied. Living material of the remaining species was not available. However, two of them (*Impatiens lotteri* and *I. kivuensis*) do not co‐occur with other species of the complex. Only the range of *Impatiens superglabra* overlaps slightly with the upper range of *Impatiens gesneroidea*. Both species share a bird pollination syndrome. Plant material was taken from plants collected in the mountain rainforests of Burundi, the Democratic Republic of the Congo and Rwanda and cultivated in the Botanical Gardens of Bonn University in Germany. Herbarium vouchers of all accessions are stored in BONN herbarium (Thiers, 2014). Several accessions per species were analyzed wherever possible. However, due to the rarity of some species or the inaccessibility of the populations this was not possible for all species. The number of accessions (= genetic plant individuals) per species is documented in Table 1.

**TABLE 1:** Fruit set of autonomous self‐pollination tests based on the observation of 20 flowers per accession

Species name	Accession no.	Fruit set of autonomous self‐pollination (%)
I. gesneroidea*	32578	0
*I. elwiraurszulae*	39568	10
*I. elwiraurszulae*	39568	10
*I. ludewigii*	17207	0
*I. ludewigii*	37954	0
*I. ludewigii*	39660	0
I. lutzmannii*	33486	0
*I. purpureoviolacea*	12079	15
I. purpureoviolacea*	36240	5
*I. purpureoviolacea*	36259	5
*I. purpureoviolacea*	37386	5
I. urundiensis*	35170	15
I. versicolor*	34558	0
*I. × troupinii*	37754	15
I. burtonii*	11528	0

Note

Data of species indicated with a * were taken from Lozada‐Gobilard et al. (2019).

### 2.2 Autonomous self‐pollination tests and pollination syndromes

Autonomous self‐pollination tests were conducted to test whether individual species of the *Impatiens purpureoviolacea* complex depart from the common pollinator dependency in most African *Impatiens* species (Lozada‐Gobilard et al., 2019), which would represent a strong mechanism preventing hybridization. To test for the proportion of fruits that develop without pollinator activity, 20 flowers each of all accession of the *Impatiens purpureoviolacea* complex as well as of the hybrid *I*. *×* *troupinii* were marked in a pollinator‐free greenhouse. Only fruits containing at least one well‐developed seed were counted as successfully developed for the fruit set.

Additionally, manual self‐pollinations were conducted for ten flowers of the wild‐collected *I*. *×* *troupinii* to test whether seeds resulting from self‐pollinations are viable. The 26 seeds resulting from these pollinations were sown out to test whether they are able to germinate and form adult plants.

Based on their pollination syndrome, most species of the *Impatiens purpureoviolacea* complex included into this study are pollinated by butterflies and bees. However, a single species, *Impatiens gesneroidea*, is probably pollinated by birds, which may act as a prezygotic mechanism preventing the hybridization with insect‐pollinated species. The categorization of pollination syndromes was taken from Abrahamczyk et al. (2017).

### 2.3 Species distribution and habitat type

The species of the *Impatiens purpureoviolacea* complex are all endemics to the northwestern Albertine Rift with some narrow endemics occurring in small elevation zones with specific habitats (Fischer et al., 2021). Several species show overlapping distribution ranges. The distributions of the species of the *Impatiens purpureoviolacea* complex as well as their habitat types were mapped by Eberhard Fischer, the taxonomic specialist of the group based on a current revision (Fischer et al., 2021), own observations in the field and the current margins of the mountain forests. If two species occur not in the same place but close to each other, with a distance of ≤2000 m, a distance bees and butterflies can fly (Araújo et al., 2004; Townsend & Levey, 2005) we treated them as geographically close, having the theoretical possibility for pollen transfer and thus to hybridize.

### 2.4 Chromosome counts

We aimed to count chromosome numbers of all species of the *Impatiens purpureoviolacea* complex to be able to reconstruct its chromosome evolution and assess its importance as a postzygotic mechanisms preventing hybridization. However, due to chromosome structure and size chromosome counts were impossible for *I*. *elwiraurszulae* and *I*. *versicolor*. The numbers of chromosomes were counted in metaphase plates, which were obtained from actively growing root tips from pot‐cultivated plants. For chromosome preparation, we used a protocol according Pijnacker and Ferwerda (1984) and Belyayev et al. (2018) with minor modification: Fresh root tips were pretreated in saturated solution of para‐dichlorobenzene at room temperature for 4 h and fixed in fresh solution of pure ethanol and glacial acetic acid (3:1) for 24 h. The fixed material was stored in fixative solution at −24°C until use. Excised roots were rinsed in double distilled water (ddH_2_O thereafter; 2 × 5 min.) and citric buffer (10 mM sodium citrate, pH 4.8, 1 × 5 min.). After that, roots were incubated for 4 h at 37°C in humid chamber in 0.3% (w/v) enzymatic solution [0.3% (w/v) cellulase, 0.3% (w/v) cytohelicase, and 0.3% (w/v) pectolyase Sigma St. Louis, MO, USA, in 10 mM citric buffer]. After digestion, root tips were transferred into the ddH_2_O and kept on ice (4°C). Chromosomes were prepared by the smear method (Pijnacker & Ferwerda, 1984). Individual root tips were transferred on clean slides and stirred by needle in 40 µl of 75% acetic acid on a warm plate (49°C) for 3 min, fixed in 300 µl fixative solution [pure ethanol and glacial acetic acid (3:1)], washed in pure ethanol, and air‐dried. Metaphases plates and chromosome counts were checked and photographed by Zeiss Axio Imager.Z2 microscope system. Photographs were prepared using Adobe Photoshop version 21.1.3.

### 2.5 Flow cytometry

To find out whether the species of the *Impatiens purpureoviolacea* complex differ in genome size, we used flow cytometry (FCM) to measure it. Nuclear DNA 2C‐values (monoploid genome sizes) were estimated using propidium iodide FCM. Each sample preparation followed the two‐step procedure (Otto, 1990). One cm^2^ of young and intact fresh leaf tissue and internal standard was mixed and chopped with a sharp razor blade in 0.5 ml of ice‐cold Otto I buffer (0.1 M citric acid, 0.5% Tween‐20). The nuclear suspension was filtered through a nylon mesh (42 μm pore size) into a plastic tube. After incubation (30 min at room temperature), 1 ml of Otto II buffer (0.4 M Na_2_HPO_4_·12H_2_O) supplemented with propidium iodide (at a final concentration 50 µl/ml), RNase IIA (50 µl/ml), and 2‐mercaptoethanol (2 µl/ml) were added. The samples were incubated for 5 min at room temperature. Fluorescence intensity of 5000 particles was recorded on a Partec Cyflow instrument (Partec GmbH) equipped with a 532 nm solid‐state laser (Cobolt Samba 100 mW, Cobolt). Each plant was re‐analyzed at least three times on different days if possible. For each run, we counted 5000 nuclei. Outlying values were discarded when between‐day variation (max./min. value) exceeded 2%. In that case, the sample was re‐measured. According to DNA content variation, *Solanum pseudocapsicum* (2C = 2.59 pg, Temsch et al., 2010), *Bellis perennis* (2C = 3.46, Doležel et al., 2007), or *Pisum sativum* “Ctirad” (2C = 8.76 pg, Doležel et al., 1998) were used as internal reference standards in order to minimize standard‐to‐sample peak ratio and thus avoid potential nonlinearity of FCM measurements.

### 2.6 Phylogenetic analysis

We generated a phylogenetic tree to reconstruct the chromosome evolution of the *Impatiens purpureoviolacea* complex. Sequence data of chloroplast *atpB*‐*rbcL* and nuclear *ImpDEF1* and *ImpDEF2* were obtained from earlier phylogenetic and evolutionary studies on *Impatiens* (e.g., Janssens et al., 2009; Fischer et al., 2021, Table S1 Appendix S1). Alignment of the sequences was carried out using the software program MAFFT (Katoh et al., 2002) with starting parameters: E‐INS‐i algorithm, 100PAM/k = 2 scoring matrix, gap open penalty of 1.3, and offset value of 0.123. Subsequent to automatic alignment with MAFFT, a manually check was performed in Geneious Prime 2020 (Biomatters). Putative incongruence between chloroplast and nuclear datasets was assessed using the hard vs. soft incongruence approach. Following this method, data matrices were visually inspected, by searching for conflicting relationships supported by a maximum‐likelihood bootstrap support value ≥70 (Johnson & Soltis, 1998; Pirie, 2015). For this, Maximum Likelihood (ML) trees of each data matrix were created using the RAxML search algorithm (Stamatakis et al., 2005) under the GTRGAMMA + I approximation of rate heterogeneity for *ImpDEF1* and GTRGAMMA for *ImpDEF2* and *atpB*‐*rbcL*.

Best‐fit nucleotide substitution models for the plastid and nuclear datasets were selected by jModelTest 2.1.4. under the Akaike Information Criterion (AIC; *ImpDEF1*: GTR + I + G, *ImpDEF1* and *atpB*‐*rbcL*: GTR + G; Posada, 2008). Since no supported topological conflict was detected among the individual gene trees (not shown), an ultrametric tree was constructed using a concatenated dataset using BEAST 1.10.1 (Suchard et al., 2018). BEAUti was used to configure the xml‐file used as input for the BEAST analysis, applying following settings; a lognormal relaxed clock model, enforcement of the “estimate” option in the clock model, a Birth‐Death Incomplete Sampling process Tree Prior and a random starting tree. All other settings were default. The analysis ran for 10,000,000 generations, sampled every 2000th generation. TRACER v.1.6 (Rambaut et al., 2014) was used to evaluate the effective sampling size of the posteriors. A maximum clade credibility (MCC) tree was calculated using TreeAnnotator v.1.10.1. (Suchard et al., 2018).

### 2.7 Statistical analysis

We conducted a *t*‐test on the genome sizes of the species of the *Impatiens purpureoviolacea* complex with 16 vs. 32 chromosomes. The analysis was conducted in R v 3.4.3 (R Development Core Team, 2017).

### 2.8 Ancestral chromosome reconstruction

To reconstruct ancestral haploid chromosome numbers and infer the type of chromosome number transitions, we used ChromEvol v. 2.0 (Glick & Mayrose, 2014). This likelihood‐based method analyses the numbers of polyploidization and dysploidization events along each branch of a phylogeny. Based on the distribution of chromosome numbers in the phylogeny, it tests several different models to estimate which of them explains the variation in chromosome numbers best. Generating 10,000 simulations the models are fitted to the data and the best model is chosen applying the AIC. We used a phylogram as well as an ultrametric phylogeny to infer ancestral chromosome numbers. Additionally, we calculated both scenarios including and excluding the inferred chromosome numbers of *I*. *elwiraurszulae* and *I*. *versicolor*.

## 3 RESULTS

### 3.1 Reproduction

All species of the *Impatiens purpureoviolacea* group are largely dependent on pollinators for seed production. Fruit set induced by autonomous selfing only ranges from 0 to 15% (Table 1). *Impatiens* *×* *troupinii* is able to form viable seeds by autogamous and manual selfing. Fruit set of the autogamous treatment is 15%, while with manual self‐pollination it is 60%. The 26 seeds (mean 4.3 ± 2.9 per fruit) resulting from manual self‐pollinations were sown out and developed to 15 adult but not very robust plants with pale pink flowers, of which a handful survived until flowering.

### 3.2 Prezygotic mechanisms preventing hybridization

**FIGURE 2:**
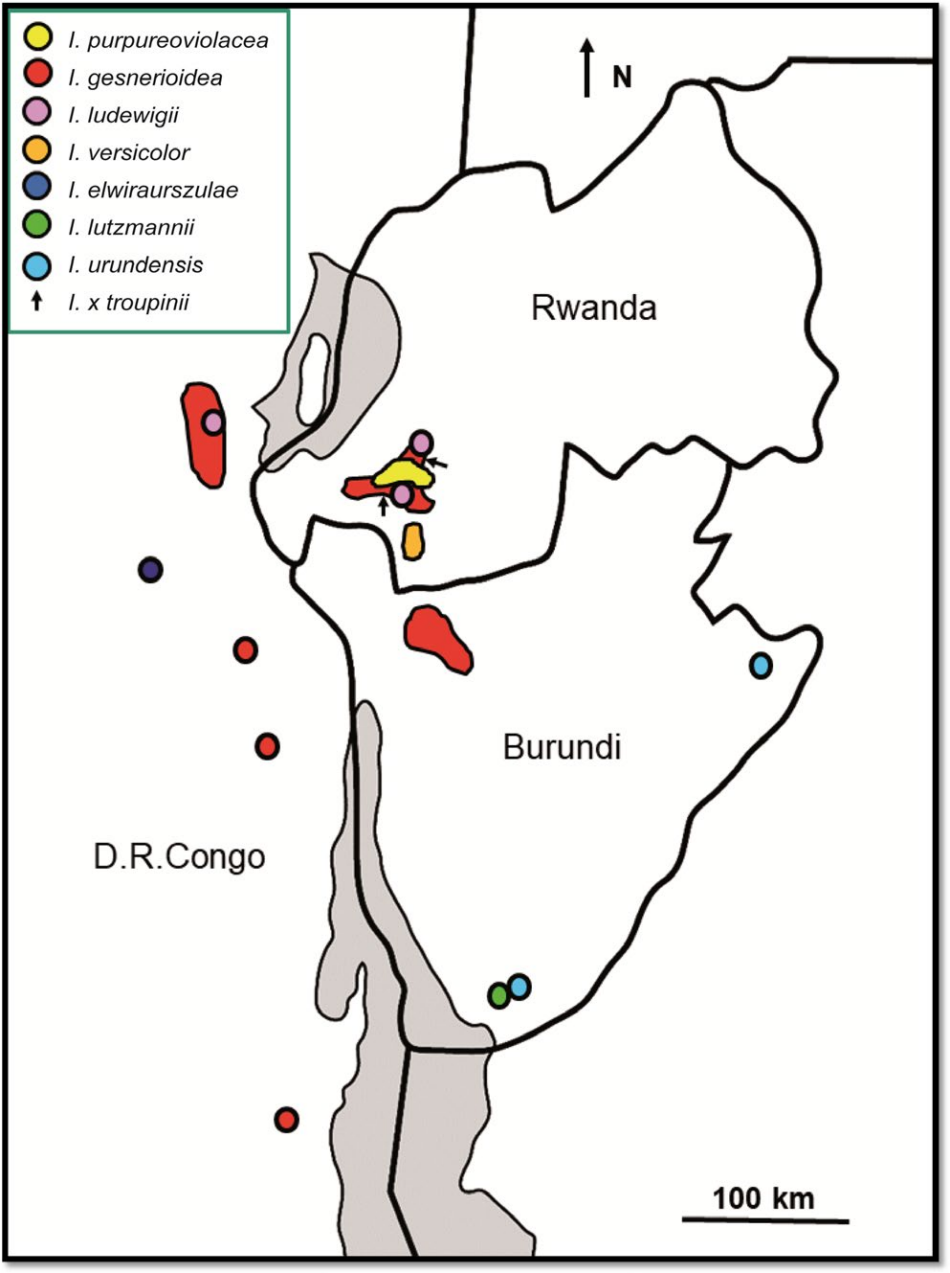
Distribution map of the species of the *Impatiens purpureoviolacea* complex analyzed in this study

Several of the seven species from the *Impatiens purpureoviolacea* complex included into this study show overlapping distribution ranges (Table 2; Figure 2): *Impatiens gesneroidea* occurs sympatrically with *I*. *purpureoviolacea* and *I*. *ludewigii*, while the latter two occur geographically close, within a range of ≤2000 m between each other and share the same habitat type. Furthermore, *I. urundiensis* and *I*. *lutzmannii* occur close to each (within a distance of ≤2000 m between each other) other but in different habitat types. Some of the sympatric/geographically close species have the same pollination syndrome (butterfly & bee), for example, *Impatiens purpureoviolacea* and *I*. *ludewigii* or *I. urundiensis* and *I*. *lutzmannii*. Other sympatric/geographically close species display different syndromes, for example, *Impatiens gesneroidea* (bird) and *I*. *purpureoviolacea* and *I*. *ludewigii* (butterfly/bee), respectively.

**TABLE 2:** Prezygotic mechanisms possibly preventing hybridization (distribution, habitat type, and pollination syndrome) in the species of the *Impatiens purpureoviolacea* complex

Species	Sympatric/geographically close	Habitat type	Elevation (m)	Pollination syndrome
*I. elwiraurszulae*	B	Lower montane rainforest	1100	Moth
*I. gesneroidea*	A	Montane rainforest	2260–2750	Bird
*I. ludewigii*	A	Montane rainforest	1700–2300	Butterfly/bee
*I. lutzmannii*	C	Montane rainforest	2180	Butterfly/bee
*I. purpureoviolacea*	A	Montane rainforest	1900–2540	Butterfly/bee
*I. urundiensis*	C	Gallery forest in grassland	1900–2000	Butterfly
*I. versicolor*	D	Montane rainforest	1800–1900	Butterfly/bee
*I. × troupinii*	A	Montane rainforest	2250–2450	Bird/bee

Note

Identical letters indicate sympatric/geographically close species. Information distribution, habitat, and elevation were taken from Fischer et al. (2021), and data on pollination syndromes were taken from Abrahamczyk et al. (2017).

### 3.3 Mechanisms preventing hybridization after pollination

Genome sizes of 17 accessions (= genetic individuals) from the *Impatiens purpureoviolacea* complex, including seven out of ten species and one natural hybrid, were measured (Table 3). Genome sizes of the species of the *Impatiens purpureoviolacea* complex with 2*n* = 16 range from 3.66 to 4.65, whereas the genome sizes of the species with 2*n* = 32 range from 7.51 to 9.45. Within the *Impatiens purpureoviolacea* complex, genome sizes differ significantly between species with 2*n* = 16 and others with 2*n* = 32 (*t*‐test, *t* = 8.65, *p* = .0001). No overlap of genome sizes exists between both groups (Table 1). Additionally, genome size was analyzed for one outgroup species (*I. rubromaculata*: 2*n* = 16; genome size = 1.127).

**TABLE 3:** Postpollination mechanisms possibly preventing hybridization (genome size and chromosome numbers) in the species of the *Impatiens purpureoviolacea* complex

Species	Accession number	DNA 2C‐value (pg)	Chromosome number (2*n*)	Source	Sympatric/geographically close species
*I. gesneroidea*	32578	9.454	32	Own data	A
*I. elwiraurszulae*	39658	9.011	–	Own data	B
*I. elwiraurszulae*	39659	9.24	–	Own data	B
*I. ludewigii*	17207	4.65	16	Own data	A
*I. ludewigii*	37751	4.611	–	Own data	A
*I. ludewigii*	37954	4.581	16	Own data	A
*I. ludewigii*	39660	4.661	–	Own data	A
*I. lutzmannii*	33486	3.656	16	Own data	C
*I. purpureoviolacea*	12079	7.972	–	Own data	A
*I. purpureoviolacea*	36240	7.575	–	Own data	A
*I. purpureoviolacea*	36259	7.517	32	Own data	A
*I. purpureoviolacea*	37386	7.951	32	Own data	A
*I. purpureoviolacea*	37752	9.351	32	Own data	A
*I. purpureoviolacea*	37753	7.856	–	Own data	A
*I. urundiensis*	35170	7.583	32	Own data	C
*I. versicolor*	34558	4.57	–	Own data	D
*I. × troupinii*	37754	8.619	32	Own data	A
*I. assurgens*	–	–	10	Gill and Chinnappa (1977)	
*I. burtonii*	–	–	14, 16	Jones and Smith (1966), Gadella (1977, 1982)	
*I. digitata*	–	–	20	Gill and Chinnappa (1977)	
*I. meruensis*	–	–	16	Oginuma and Tobe (1991)	
*I. rubromaculata*	36245	1.127	16	Jones and Smith (1966)	
*I. ulugurensis*	–	–	16	Gill and Chinnappa (1977)	

Note

Identical letters indicate sympatric/geographically close species. Only chromosome numbers of outgroup species have been taken from other publications.

**FIGURE 3:**
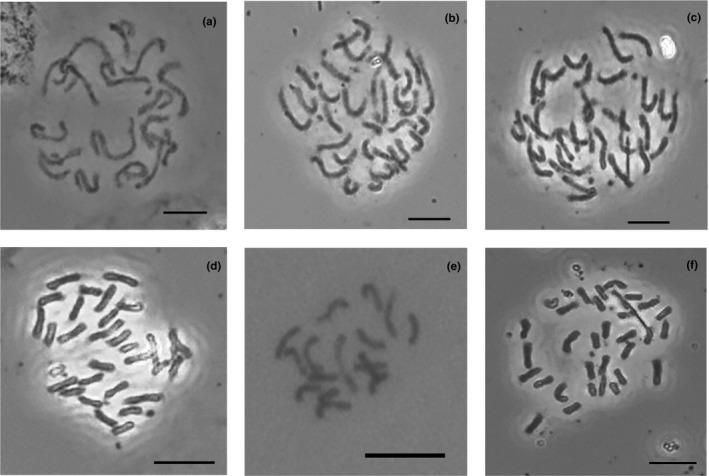
Chromosome photographs of the *Impatiens purpureoviolacea* complex: (a) *I*. *lutzmannii* (2*n* = 16), (b) *I. urundiensis* (2*n* = 32), (c, d) *I*. *purpureoviolacea* (2*n* = 32), (e) *I. ludewigii* (2*n* = 16), (f) *I. gesneroidea* (2*n* = 32). Scale bars indicate 10 µm

**FIGURE 4:**
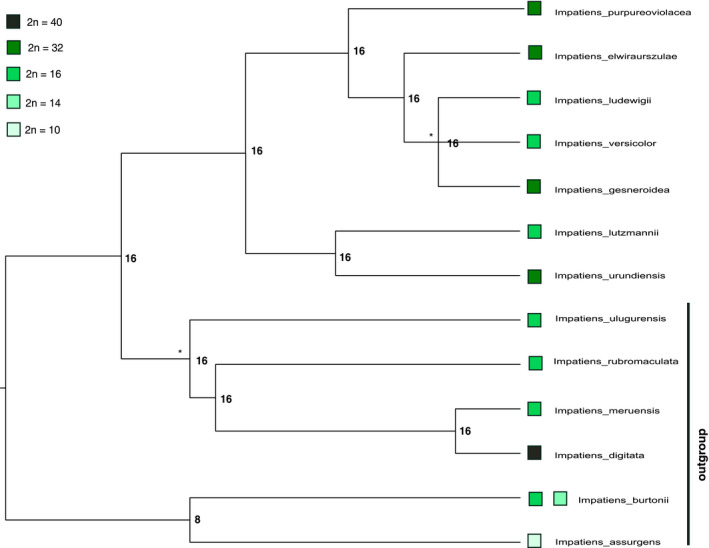
Ancestral reconstructions of chromosome numbers along an ultrametric tree of the *Impatiens purpureoviolacea* complex. Node labels indicate ancestral chromosome numbers. * indicate nodes with a support of <65%. Chromosome numbers of *Impatiens elwiraurszulae* and *I*. *versicolor* have been inferred from genome sizes. Including or excluding these species from the analysis does not influence the results of inferred ancestral chromosome numbers

Chromosome numbers were counted for nine accessions, including five species and one natural hybrid (Table 3). Additionally, chromosome numbers of six closely related outgroup species were taken from literature (Table 1). The chromosome numbers of the closely related outgroup species are diverse, ranging from 2*n* = 10 to 2*n* = 20 with a majority of species with 2*n* = 16 (Figures 3 and 4). Within the *Impatiens purpureoviolacea* complex, most species have chromosome numbers of 2*n* = 32. This is probably also the case for *I*. *elwiraurszulae*, based on its genome size. In the early branching clade of the *I*. *purpureoviolacea* complex, only *I*. *lutzmannii*, which is sister to *I. urundiensis* and *I*. *ludewigii* in the terminal clade, have chromosome numbers of 2*n* = 16. For *Impatiens versicolor*, for which no chromosome number was determined, the genome size also indicated a chromosome number of 2*n* = 16. Sympatric/geographically close species either have identical chromosome numbers, for example, *Impatiens purpureoviolacea* and *I*. *gesneroidea* (2*n* = 32) or differ in chromosome numbers, for example, *I*. *ludewigii* and *I*. *gesneroidea* or *I*. *lutzmannii* and *I. urundiensis* (2*n* = 16 and 2*n* = 32, respectively).

A phylogram of the *Impatiens purpureoviolacea* complex with branch support is presented in Figure S1 (Appendix S1) and shows similar evolutionary relationships as delineated in previous phylogenetic studies on *Impatiens* (e.g., Fischer et al., 2021; Janssens et al., 2009). In general, the overall topology is well supported, except for the clade containing the polytomy of *I. ludewigii*, *I. versicolor*, and *I*. *gesneroidea*, which only gains low support. The ChromEvol analysis showed that a model allowing dysploidy and genome duplication (polyploidy) is most appropriate for our data, independent if a phylogram or an ultrametric phylogeny was used, respectively, we included the inferred chromosome numbers of *Impatiens elwiraurszulae* and *I*. *versicolor* into the analysis or not. The ancestral chromosome number reconstruction indicated 2*n* = 8 for the ancestors of the early branching outgroup clade. For all other nodes except the stem node of the *I*. *purpureoviolacea* complex, 2*n* = 16 was reconstructed, independent which tree was used or if we included the inferred chromosome numbers of *Impatiens elwiraurszulae* and *I*. *versicolor* or not (Figure 3). The analysis using the ultrametric tree also revealed 2*n* = 16 for the stem node of the *I*. *purpureoviolacea* complex. However, using the phylogram the ancestral chromosome reconstruction revealed 2*n* = 8 for this node. Thus, all species of the *I*. *purpureoviolacea* complex with chromosome number of 2*n* = 32 represent polyploidization events, and at least four events of polyploidization seem to have occurred in its evolution.

## 4 DISCUSSION

To date, this is the only study in the species‐rich genus *Impatiens* as well as one of the first studies in flowering plants in which a multidisciplinary approach is applied where reproductive, cytological, geographical, and phylogenetic information is combined to investigate pre‐ and postzygotic mechanisms that may prevent hybridization within an entire clade. Bridging the gap between micro‐ and macro‐evolution, we are able to document by which mechanisms the diversity of the small *Impatiens purpureoviolacea* clade may have evolved. However, our approach may be applicable to explain the amazing diversity not only in the genus *Impatiens* but in many other species‐rich genera with co‐occurring species as well. Ideally, future studies using a similar approach should have a balanced design analyzing the traits of a number of individuals per species and include all species of a clade. Further, a species distribution model may be applied to analyze possible range overlaps if sufficient distribution data of the species exist.

Most species of the *Impatiens purpureoviolacea* complex (17 out of 21 species pairs; Table 4) are separated by large geographical distances between their ranges in the topologically heterogeneous landscape of the Albertine Rift. Only the closely related *Impatiens gesneroidea*, *I*. *ludewigii* and *I*. *purpureoviolacea* occur sympatrically/geographically close in Rwanda and the adjacent Democratic Republic of the Congo, while the sister species *I. lutzmanii* and *I. urundiensis* occur geographically close in Burundi (Fischer et al., 2021). These self‐compatible but largely pollinator‐dependent species are surprisingly variable in their habitats as well as in reproductive and cytological traits, which reflects the distribution of traits in the entire genus (Abrahamczyk et al., 2017; Jeelani et al., 2010; Lozada‐Gobilard et al., 2019; Song et al., 2003). However, this trait diversity may have evolved as an adaptation to local conditions as well as a mechanism to prevent hybridization.

**TABLE 4:** Overview of mechanisms potentially preventing hybridization between the individual species of the *Impatiens purpureoviolacea* complex; c = chromosome numbers, g = geography, h = habitat, s = pollination syndrome; (c) = chromosome number inferred from genome size

	*I. gesneroidea*	*I. ludewigii*	*I. lutzmannii*	*I. purpureoviolacea*	*I. urundiensis*	*I. versicolor*
*I. elwiraurszulae*	g, h, s	(c), g, h, s	g, h, s	g, h, s	g, h, s	(c), g, h, s
*I. gesneroidea*		c, s	c, g, s	s	g, h, s	(c), g, s
*I. ludewigii*			g	c	c, g, h	g
*I. lutzmannii*				c, g	c, h	g
*I. purpureoviolacea*					g, h	g
*I. urundiensis*						g, h

Chromosome numbers and genome sizes are highly correlated to each other in the *Impatiens purpureoviolacea* complex. The reconstructions for the crown node (and using the ultrametric tree also for the stem node) of the clade indicated 2*n* = 16 chromosomes, the most common number of chromosomes in *Impatiens* (Song et al., 2003). This is also true for all other nodes within the *I*. *purpureoviolacea* complex. Therefore, four independent polyploidization events occurred within the clade (*I. urundiensis*, *I. purpureoviolacea*, *I*. *elwiraurszulae*, and *I. gesneroidea*). Since we see little morphological variability in chromosome structure and do not have any evidence for a combination of hybridization and polyploidization, we assume that all species are auto‐polyploids. All of these polyploidization events took place in sympatry with a diploid species from the *Impatiens purpureoviolacea* complex. In addition, evolutionary changes of a second ecological trait (habitat and/or pollination syndrome) may act as a further mechanism preventing hybridization as well (Table 4). Just *Impatiens ludewigii* and *I*. *purpureoviolacea* occur geographically close to each other and only differ in chromosome numbers; however, the contact zone of both species is small. Such combinations of cytological and ecological mechanisms that may prevent hybridization have been reported repeatedly in a range of more or less species‐rich genera with young radiations, for example, in *Achillea* (Asteraceae), *Silene* (Caryophyllaceae), *Chamaenerion* (Onagraceae), or *Houstonia* (Rubiaceae; e.g., Glennon et al., 2012; Husband & Sabara, 2004; Karrenberg et al., 2019; Ramsey, 2011). These mechanisms often separate populations with different ploidy levels within the same species as well as between closely related species.

The only known natural hybrid of the *Impatiens purpureoviolacea* complex, *I*. *×* *troupinii* represents rare crossing events of the two auto‐polyploids *I*. *purpureoviolacea* and *I*. *gesneroidea* (both 2*n* = 32). *Impatiens* *×* *troupinii* has been observed in the wild only a few times since the early 1980s (Fischer et al., 2021). While *I*. *purpureoviolacea* has a butterfly/bee syndrome, *I*. *gesneroidea* has a bird pollination syndrome. The flowers of *I*. *×* *troupinii* show characters of both parental species. *I*. *×* *troupinii* is self‐fertile; however, the young plants resulting from self‐pollinated seeds of *I*. *×* *troupinii* are not very viable, which may indicate postzygotic mechanisms preventing hybridization. Additionally, the plants growing from self‐pollinated seeds of *I*. *×* *troupinii* show pale pink flowers, which may be less attractive for pollinators. These observations may explain why no larger hybrid populations exist. Similar observations have been reported for *I. ×* *lateritia*, a natural hybrid between the bird‐pollinated *kilimanjari* ssp. *kilimanjari* and the insect‐pollinated *I. pseudoviola* (Grimshaw & Grey‐Wilson, 1997). However, the hybrid of the second subspecies of *Impatiens kilimanjari* (*I*. *kilimanjari* ssp. *pocsii*) and *I. pseudoviola—I. ×* *kaskazini—*is vigorously growing and relatively common, but occurs only at anthropogenically disturbed places in the forest (Grimshaw & Grey‐Wilson, 1997).

In conclusion, the rare occurrence of *I*. *×* *troupinii* and *I. ×* *lateritia* and the occurrence of *I. ×* *kaskazini* only at anthropogenically disturbed places indicate that different pollination syndromes and habitat are strong but no absolute mechanisms potentially preventing hybridization in *Impatiens*. However, under natural conditions the combination of these mechanisms probably works well prohibiting hybridization in *Impatiens*. Similar combinations of mechanisms preventing hybridization may occur in other species‐rich genera with co‐occurring, closely related species as well.

## CONFLICT OF INTEREST

The authors declare no competing interests.

## AUTHOR CONTRIBUTION

**Stefan Abrahamczyk:** Conceptualization (lead); Formal analysis (lead); Project administration (lead); Writing‐original draft (lead). **Michaela Jandova:** Methodology (supporting); Writing‐review & editing (supporting). **Zuzana Liblova:** Formal analysis (supporting); Writing‐original draft (supporting). **Steven Janssens:** Formal analysis (supporting); Writing‐original draft (supporting). **Tomas Dostalek:** Formal analysis (supporting); Writing‐original draft (supporting). **Norbert Holstein:** Formal analysis (supporting); Writing‐original draft (supporting). **Eberhard Fischer:** Conceptualization (supporting); Resources (supporting); Writing‐original draft (supporting).

## Supporting information

Appendix S1

## Data Availability

All data are presented in the main body of the article and in the Appendix S1.
