# Supplementary material for: Pre‐ and postzygotic mechanisms preventing hybridization in co‐occurring species of the Impatiens purpureoviolacea complex
Source: Ecol Evol. 2021 Nov 24;11(23):17485–95. doi: 10.1002/ece3.8382 (PMC8668770; doi:10.1002/ece3.8382)
Supplement: Supplementary file 1 — Appendix S1 [file ECE3-11-17485-s001.docx]

**Appendix**

Table S1: List of species names, voucher information and GenBank accession numbers of plant material used in this study

| **Taxon** | **Voucher** | ***ImpDEF1*** | ***ImDEF2*** | ***atpB-rbcL*** |
| --- | --- | --- | --- | --- |
| *I. ludewigii* | Fischer 14500 (KOBL) | MT612876 | MT612859 | MT612889 |
| *I. versicolor* | Fischer 13390 (KOBL) | MT612874 | - | MT612902 |
| *I. gesneroidea* | Fischer 11021 (KOBL) | MT612877 | MT612867 | MT612892 |
| *I. elwiraurszulae* | Dumbo & Dumbo s.n. (KOBL) | MT612879 | MT612864 | - |
| *I. purpureoviolacea* | Fischer 8093 5KOBL) | MT612883 | MT612858 | M612897 |
| *I. lutzmannii* | Fischer 13002 (KOBL) | MT612873 | MT612855 | MT612900 |
| *I. urundiensis* | Fischer 13301 (KOBL) | MT612871 | MT612854 | MT612905 |
| *I. meruensis* ssp. *meruensis* | Knox 3328 (BR) | FJ826714 | FJ826767 | FJ826662 |
| *I. digitata* ssp. *digitata* | Knox 3653 (BR) | FJ826689 | FJ826744 | FJ826638 |
| *I. rubromaculata* ssp. *rubromaculata* | Knox 3667 (BR) | EU723721 | EU723727 | EU723714 |
| *I. ulugurensis* | Knox 3554 (BR) | FJ826732 | FJ826786 | FJ826678 |
| *I. burtonii* var. *burtonii* | Knox 2803 (BR) | FJ826685 | FJ826740 | FJ826885 |
| *I. assurgens* | Dessein 720 (BR) | FJ826681 | FJ826735 | FJ826629 |


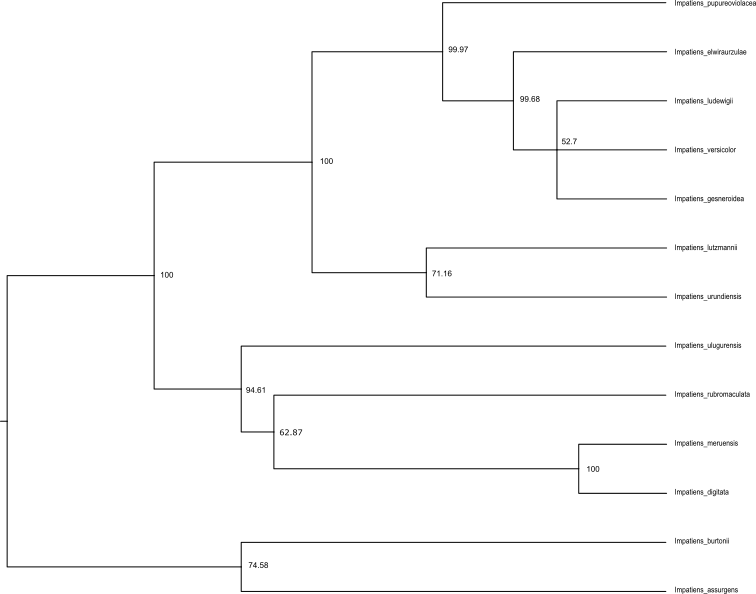


Figure S1: Likelihood phylogeny of the *Impatiens purpureoviolacea* complex. Numbers at branch nodes indicate bootstrap support.
